# Comparing the Prevalence of Sleep Disorders Among Underweight, Normal, Overweight, and Obese Adults in Riyadh, Saudi Arabia

**DOI:** 10.7759/cureus.58858

**Published:** 2024-04-23

**Authors:** Mohammed A AlAteeq, Meshael M Alghaihab, Lujain K Marghlani, Lenah A Shamsaddin, Remaz K Alghamdi, Maha A Alfadley

**Affiliations:** 1 Family Medicine and Primary Health Care, Ministry of the National Guard - Health Affairs, Riyadh, SAU; 2 Family Medicine and Primary Health Care, King Abdullah International Medical Research Center, Riyadh, SAU; 3 Family Medicine and Primary Health Care, King Saud bin Abdulaziz University for Health Sciences, Riyadh, SAU; 4 College of Medicine, King Saud bin Abdulaziz University for Health Sciences, Riyadh, SAU

**Keywords:** chronic conditions, obesity, insomnia, narcolepsy, obstructive sleep apnea

## Abstract

Background

Sleep disorders are prevalent worldwide and can have a negative impact on physical and psychological well-being. Numerous studies have explored the reciprocal connection between obesity and sleep disorders. This study aimed to compare the prevalence of sleep disorders among underweight, normal, overweight, and obese adults in Riyadh, Saudi Arabia.

Methods

This cross-sectional study was conducted on 378 adults visiting primary healthcare centers in Riyadh, Saudi Arabia, from August to November 2022. Data were collected using a self-administered questionnaire that included a section for demographic data and the *SLEEP-50 *questionnaire in both English and Arabic languages.

Results

Most of the participants were aged between 25 and 34 years (37.6%), 79.1% were females and 59.5% were either overweight or obese. Most participants (78.3%) had at least one sleep disorder, with narcolepsy being the most frequent disorder (65.1%), and 23% had two combined sleep disorders. Obese and overweight patients were significantly more likely to have sleep disorders (p=0.011), and obese patients were more likely to have all sleep disorders (p=0.049).

Conclusion

The prevalence of sleep disorders, namely narcolepsy and insomnia, is high among adults in Riyadh, Saudi Arabia. Moreover, sleep disorders are significantly associated with obesity. Evaluation and management of sleep disorders in clinical settings among patient with overweight or obese is important to improve their quality of life and to prevent physical and psychological complications.

## Introduction

Sleep disorders encompass a wide range of conditions that disrupt sleep quality, duration, or schedule, including insomnia, obstructive sleep apnea (OSA), and narcolepsy. These disorders can have detrimental effects on both physical and psychological well-being, potentially impairing neurocognitive processes and increasing the risk of mood fluctuations and depression [1,2].

Sleep disorders are prevalent worldwide. For example, a systematic review conducted in the Netherlands, the United Kingdom, and the United States estimated that one in four individuals slept less than the recommended duration for their age group. In adults aged 18 and above, the prevalence of insomnia and poor sleep quality was particularly high in the United States. Women were found to have shorter sleep duration and poorer sleep quality compared to men [3]. In Saudi Arabia, approximately one in three adults had a short sleep duration, with insomnia being more common among females and the elderly [4,5]. Additionally, the prevalence of OSA was significantly higher in males, married individuals, obese individuals, and those above the age of 30 [6].

Maintaining a healthy weight is crucial for overall health and is associated with the prevention and management of various conditions, including diabetes, cardiovascular disease, and sleep apnea. The prevalence of obesity and overweight in adults exceeds that of underweight individuals. In a study conducted in multiple European countries, the distribution of weight categories was approximately 2% underweight, 45% normal weight, 37% overweight, and 16% obese [7]. In Saudi Arabia, it has been reported that nearly 15% of young adults are underweight, while 24.7% of adults were classified as obese in 2020 [8,9].

The literature supports a bidirectional relationship between obesity and sleep disorders. Obesity can significantly impact the sleep-wake cycle, leading to decreased sleep quality. Conversely, sleep patterns, quality, and duration can influence weight by affecting the production of hormones that regulate hunger and appetite [10]. In Saudi Arabia, the high prevalence of poor sleep quality has been associated with an unhealthy and sedentary lifestyle, increased body fat and waist circumference, and obesity [11].

Despite the growing evidence of the complex relationship between sleep and body mass index (BMI) and their combined effects on the human body, studies comparing sleep disorders across different BMI categories are limited in Saudi Arabia due to the relatively recent emergence of sleep medicine. Furthermore, many of the existing studies have primarily focused on the relationship between sleep disorders and obesity, neglecting other BMI categories. However, a local study assessing the severity and prevalence of OSA found significantly higher rates of the disorder among obese individuals and men, in contrast to an older study where the prevalence was higher in women [12]. Another study identified a BMI of 25 or above as the only independent significant indicator associated with an increased risk of OSA [6]. Additionally, 8.5% of patients referred to sleep disorder physicians with suspected OSA were diagnosed with obesity hypoventilation syndrome [13]. The current study aimed to compare the prevalence of sleep disorders among underweight, normal-weight, overweight, and obese adults in Riyadh, Saudi Arabia.

## Materials and methods

Study design and study population

This study was an observational cross-sectional study conducted at three primary healthcare centers in Riyadh, Saudi Arabia, from August 2022 to November 2022. The three centers included the Health Care Specialty Center (HCSC), King Abdulaziz City Housing Clinics (Iskan), and the National Guard Comprehensive Specialized Clinic (NGCSC). These centers provide primary healthcare services for pediatric and adult patients with various medical conditions. The total annual number of visits to these centers is approximately 350,000.

Inclusion criteria

The study included adults, both Saudi and non-Saudi, aged between 18 and 65 years, of both genders, who visited the three primary healthcare centers during the study period.

Exclusion criteria

Certain groups were excluded from the study, including patients with sleep disorders caused by organic factors other than OSA (such as cardiac disease, pulmonary disease, joint disease, chronic pain, etc.), patients with mental health disorders, patients taking psychiatric medications for any indication, illiterate patients, and pregnant women.

Sample size and sampling technique

Based on a review of previous studies on the prevalence of sleep disorders in Saudi Arabia [4,5], an assumed prevalence of 50% was used for sample size estimation. With a 5% margin of error and a 95% confidence level, the estimated sample size was 377, calculated using the Raosoft calculator. A non-probability convenience sampling technique was used, whereby individuals who were available during data collection and met the inclusion criteria were approached to participate in the study.

Data collection and analysis

The main independent variables in the study included BMI, age, gender, education level, employment status, smoking status, chronic medication use, type of work shift, chronic disease status, and caffeine consumption. The outcome variables were the prevalence and types of sleep disorders. After obtaining institutional review board (IRB) approval, eligible subjects were approached by the co-investigators in the waiting area of the primary care centers during their visits. Informed consent was obtained from the participants, and data was collected using a self-administered questionnaire.

The questionnaire consisted of two parts. Part one collected demographic data, including age, gender, education level, employment status, smoking status, chronic disease status, chronic medication use, type of work shift, and caffeine consumption. Part two included the Sleep 50 questionnaire in both English and Arabic versions. The Sleep 50 questionnaire is a validated tool used to screen for seven different sleep disorders [14]. It consists of 50 questions divided into nine sections, covering various aspects of sleep disorders. Each item is scored on a four-point scale ranging from 1 (not at all) to 4 (very much).

To be considered a sleep complaint, an item needed to have a minimum score of three. A section was considered to indicate the presence of a possible sleep disorder if it had at least one sleep complaint in addition to exceeding the optimal cut-off value for that section. The cut-off values for each section varied depending on the specific sleep disorder being assessed.

The Arabic version of the Sleep 50 questionnaire was developed by the investigators and validated through a translation and back-translation process, as well as expert review and piloting with a sample of 20 participants. To minimize bias, only physical copies of the questionnaire were distributed.

Data on weight, height, and BMI were measured by the co-investigators themselves using available scales in the screening rooms. Participants were classified into different BMI categories (underweight, normal weight, overweight, and obese) based on the CDC classification. Self-reported height and weight by the participants were not accepted for this study.

Descriptive statistics were used to summarize categorical variables as counts and proportions (%). The relationship between sleep disorders and participants' socio-demographic characteristics was analyzed using the Chi-square test. The chi-square test was also used to compare the prevalence of sleep disorders among different BMI categories. A p-value less than 0.05 was considered statistically significant. Statistical analysis was performed using the Statistical Packages for Social Sciences (SPSS) version 26 (IBM Corp., Armonk, NY).

## Results

Descriptive analysis

A total of 378 patients participated in this study. Table 1 provides an overview of the socio-demographic characteristics of the participants. The majority of respondents (37.6%) were between the ages of 25 and 34, and most of them were females (79.1%). Around 69% of the participants had completed college or bachelor's degrees. More than half of the participants (52.9%) were employed, and nearly half of them (48.4%) had regular daytime work schedules. The majority of participants (82.5%) reported daily caffeine consumption.

**Table 1:** Socio-demographic characteristics of the patients (n=378)

Study variables	N (%)
Age group	
18 – 24 years	86 (22.8)
25 – 34 years	142 (37.6)
35 – 44 years	80 (21.2)
45 – 54 years	58 (15.3)
55 – 65 years	12 (03.2)
Gender	
Male	79 (20.9)
Female	299 (79.1)
Family medicine center	
Iskan	64 (16.9)
HCSC	208 (55.0)
NGCSC	106 (28.0)
Educational level	
Primary/Intermediate	21 (05.6)
High School	88 (23.3)
College/Bachelor	261 (69.0)
Postgraduate	08 (02.1)
Employment	
Employed	200 (52.9)
Unemployed	125 (33.1)
Student	53 (14.0)
Type of work shift	
Always Day Time	183 (48.4)
Always Nighttime	03 (0.80)
Alternating	14 (03.7)
None	178 (47.1)
Daily caffeine consumption	
Yes	312 (82.5)
No	66 (17.5)
Smoking	
Yes	25 (06.6)
No	345 (91.3)
Ex-smoker	08 (02.1)
BMI level	
Underweight (kg/m2)	28 (07.4)
Normal (kg/m2)	125 (33.1)
Overweight (kg/m2)	127 (33.6)
Obese (kg/m2)	98 (25.9)
Currently taking medication	
Yes	114 (30.2)
No	264 (69.8)

**Figure 1:**
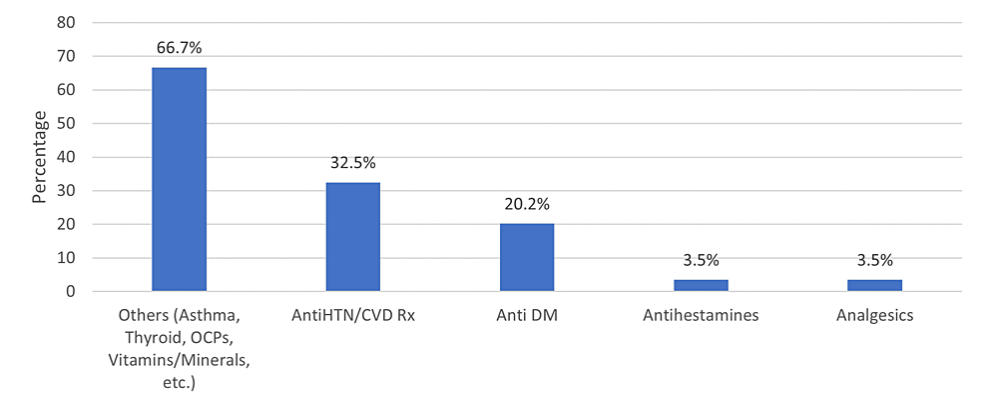
Type of medication being taken

The prevalence of smoking among the respondents was 6.6%. In terms of BMI, a significant proportion of participants were overweight (33.6%) or obese (25.9%). Additionally, 30.2% of participants reported currently taking medications. Among those taking medication, the most commonly prescribed medications were antihypertensive/cardiovascular medications (32.5%) and antidiabetic medications (20.2%) (Figure 1).

**Figure 2:**
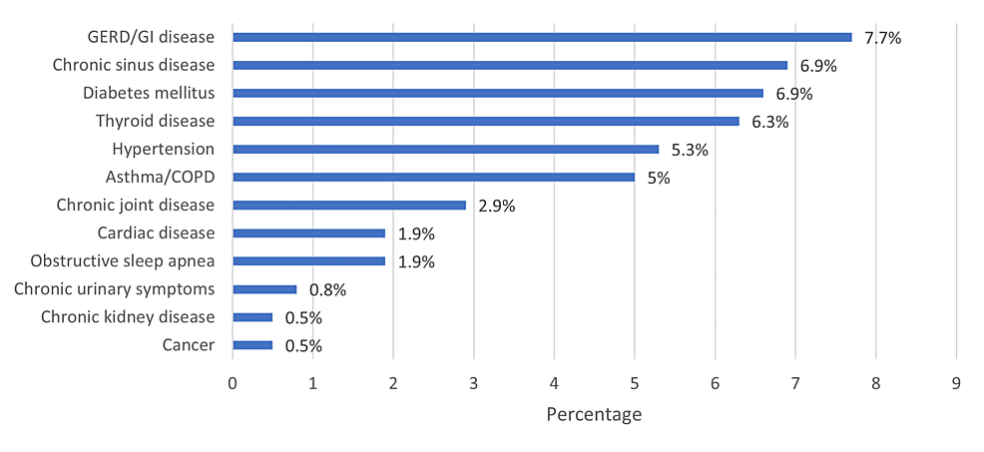
Associated chronic diseases

The most prevalent chronic diseases reported were gastroesophageal reflux/GI disease (7.7%), chronic sinus disease (6.9%), and diabetes mellitus (6.9%) (Figure 2).

Prevalence of sleep disorders

**Figure 3:**
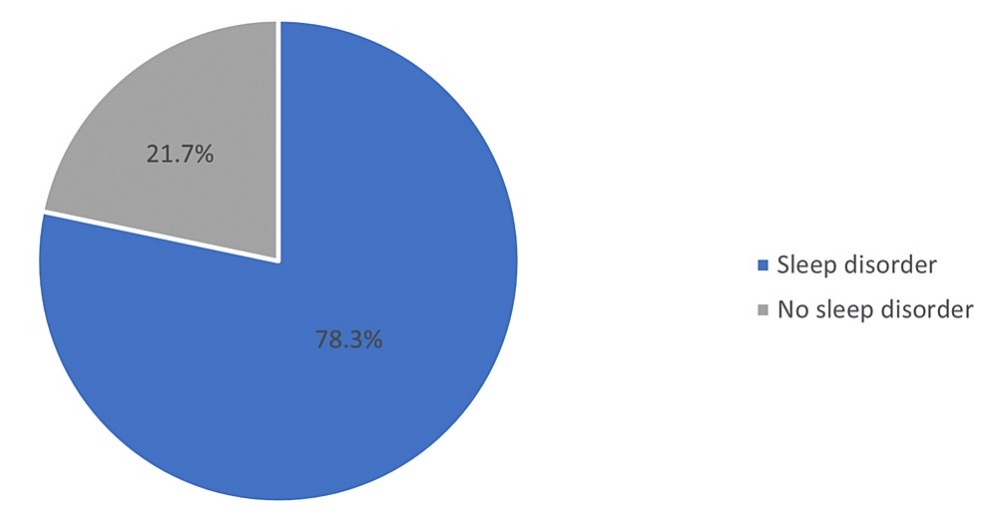
Prevalence of sleep disorders

The study found that 78.3% of participants had at least one sleep disorder (Figure 3). As shown in Table 2, the most common sleep disorders reported were narcolepsy (65.1%), insomnia (45.2%), and restless legs syndrome (31.7%). Nightmares (9.5%) and sleepwalking (4.5%) were the least prevalent disorders. Circadian rhythm disorders and OSA were reported at similar rates, approximately 18.0% and 17.7%, respectively. Out of the participants, 23% had two combined sleep disorders, 22.5% had only one sleep disorder, and 16.4% had three combined sleep disorders (Table 3).

**Table 2:** Prevalence of sleep disorders according to SLEEP-50 questionnaire

Sleep-50 domains	N (%)
Narcolepsy	246 (65.1)
Insomnia	171 (45.2)
Restless legs syndrome	120 (31.7)
Circadian rhythm disorder	68 (18.0)
Obstructive sleep apnea	67 (17.7)
Nightmare	36 (9.5)
Sleepwalking	17 (4.5)

**Table 3:** Frequencies of combined sleep disorders (n=378)

Sleep disorder	N (%)
No Sleep disorder	82 (21.7)
One sleep disorder	85 (22.5)
Two combined sleep disorders	87 (23.0)
Three combined sleep disorders	62 (16.4)
Four or more combined sleep disorders	62 (16.5)

Bivariate analysis

When analyzing the relationship between sleep disorders and socio-demographic factors, significant associations were found between sleep disorders and younger age groups (p=0.031), and unemployed/students (p=0.002). Likewise, participants with overweight or obesity were more likely to have sleep disorders (p=0.003) (Table 4).

**Table 4:** Relationship between sleep disorders and the socio-demographic characteristics of participants (n=378) *P-value has been calculated using the Chi-square test. **P-value considered significant at <0.05 level.

Factor	Sleep disorder		P-value ^*^
	Yes N (%) ^(n=296)^	No N (%) ^(n=82)^	
Age group			
<35 years	187 (63.2)	41 (50.0)	0.031 **
≥35 years	109 (36.8)	41 (50.0)	
Gender			
Male	59 (19.9)	20 (24.4)	0.380
Female	237 (80.1)	62 (75.6)	
Educational level			
High School or below	87 (29.4)	22 (26.8)	0.650
Bachelor or higher	209 (70.6)	60 (73.2)	
Employment			
Employed	144 (48.6)	56 (68.3)	0.002 **
Unemployed/Student	152 (51.4)	26 (31.7)	
BMI level			
Normal or underweight	108 (36.5)	45 (54.9)	0.003 **
Obese or overweight	188 (63.5)	37 (45.1)	
Daily caffeine consumption			
Yes	245 (82.8)	67 (81.7)	0.822
No	51 (17.2)	15 (18.3)	
Smoking			
Smoker/Ex-smoker	27 (09.1)	06 (07.3)	0.608
Non-smoker	269 (90.9)	76 (92.7)	
Currently taking medication			
Yes	94 (31.8)	20 (24.4)	0.198
No	202 (68.2)	62 (75.6)	
Associated chronic disease			
Yes	104 (35.1)	23 (28.0)	0.229
No	192 (64.9)	59 (72.0)	

However, no significant relationships were observed between sleep disorders and gender, education level, caffeine consumption, smoking, current medication use, or associated chronic diseases (all p>0.05). In the context of conducting a subanalysis on individual sleep disorders, the limited participant count within each subgroup did not yield sufficient statistical significance. However, a direct correlation was observed between the presence of sleep disorders and the occurrence of overweight or obesity, particularly in the cases of OSA and restless leg syndrome. Notably, when considering both overweight and obese subjects as a combined group, the correlation becomes more pronounced and evident. Additionally, obese patients were more likely to experience adverse effects of sleep disorders on daily functioning compared to patients with normal BMI levels (p=0.049) (Table 5).

**Table 5:** Prevalence of sleep disorders according to BMI level (n=378) *P-value has been calculated using the Chi-square test. **P-value considered significant at <0.05 level.

Sleep-50 domains	BMI level				P-value ^§^
	Underweight N (%)	Normal N (%)	Overweight N (%)	Obese N (%)	
Obstructive sleep apnea	4 (14.3)	19 (15.2)	24 (18.9)	20 (20.4)	0.727
Insomnia	16 (57.1)	56 (44.8)	49 (38.6)	50 (51.0)	0.157
Narcolepsy	20 (71.4)	83 (66.4)	74 (58.3)	69 (70.4)	0.220
Restless legs syndrome	09 (32.1)	37 (29.6)	39 (30.7)	35 (35.7)	0.795
Circadian rhythm disorder	5 (17.9)	27 (21.6)	19 (15.0)	17 (17.3)	0.589
Sleepwalking	2 (7.1)	7 (5.6)	4 (3.1)	4 (4.1)	0.601
Nightmare	4 (14.3)	13 (10.4)	7 (5.5)	12 (12.2)	0.203
Impact on daily functioning	1 (3.6)	10 (8.0)	18 (14.2)	18 (18.4)	0.049 **

## Discussion

The findings of this study indicate a high prevalence of sleep disorders among the participants. Another local study focusing on university students reported an even higher prevalence of at least one sleep disorder, reaching 73.7% [15]. However, it is important to note that university students may have higher rates of sleep disorders compared to other age groups. Similarly, a local study conducted on adults with a mean age of 42.3 years found that 78.3% of respondents reported poor sleep quality [4]. In contrast, a study on Chinese university students reported a much lower rate of sleep disorders [16]. The variability in reported rates may be attributed to differences in methodology and the age groups of the study populations.

The most commonly reported sleep disorder in the current study was narcolepsy, followed by insomnia. Similarly, two local studies reported high rates of narcolepsy. One study conducted on adult men and women healthcare workers at a tertiary hospital in Jeddah reported a prevalence of 32% [17]. Another study conducted on medical students in Makkah reported a remarkably high rate of 51.5% [15]. Both studies utilized the Sleep-50 questionnaire. Regionally, a prevalence of 7.9% of narcolepsy was reported among Jordanian medical students [18].

In comparison, the reported rates of narcolepsy across different populations were 2.92% in Europe, 5.14% in North America, 3.57% in Asia, and 2.42% among Caucasian populations [19]. This significant difference in narcolepsy prevalence estimates may be related to the accuracy of self-reporting symptoms by respondents and warrants further investigation for confirmation. It is assumed that the reported frequencies in Saudi Arabia may not precisely reflect the current prevalence of this disorder, possibly due to the complexity of symptoms and under-recognition of the diagnosis, as a considerable interval between the onset of narcolepsy symptoms and diagnosis was noted.

Additionally, the study found that nearly a quarter of the respondents had two combined sleep disorders, and a significant number of participants had multiple sleep disorders occurring concurrently. This finding was consistent with a local study where 19.9% of participants exhibited two sleep disorders [15]. However, a higher rate was reported in an Australian study, where 56% of participants demonstrated comorbid sleep conditions [20]. These results highlight the frequent co-occurrence of sleep disorders and the intricate interactions between them. Having one sleep disorder may be a risk factor for having another, and the impact on health could be worse. For example, the co-occurrence of insomnia and OSA within the same patient is associated with consistently worse health outcomes compared to individuals with neither condition nor those with either condition alone [21]. Recognizing the patterns of comorbidity among different sleep disorders can assist clinicians in formulating comprehensive treatment strategies that address the complex nature of these conditions.

Furthermore, the study revealed that the prevalence of sleep disorders was significantly higher among younger age groups, students, and unemployed individuals. This finding is consistent with previous studies that have identified young adults as a vulnerable group for sleep disorders [2,22,23]. Young adulthood is often characterized by lifestyle changes, increased stress levels, and irregular sleep patterns, which may contribute to a higher prevalence of sleep disorders among this population.

However, another local study reported a higher prevalence among the elderly, with age-adjusted prevalence rates of insomnia at 93.7%, 79.8%, and 64.2% for the elderly, middle-aged, and young groups, respectively [5]. This disparity may be explained by the smaller sample size of participants above 55 years in the current study (only 3.2%), as well as the focus on multiple sleep disorders compared to the previous study that only examined insomnia. Another study that investigated age-related differences in self-reported sleep quality using latent class analysis identified four sleep types associated with different age groups: “good sleepers” were most frequent in middle age, “inefficient sleepers” were most frequent in old age, “delayed sleepers” were most frequent in young adults, and “poor sleepers” were most frequent in old age [24].

Moreover, the study found a positive correlation between unemployment status and an increased prevalence of sleep disorders. This association has also been reported in other studies [25,26].

The prevalence of sleep disorders was also found to be higher among overweight and obese individuals. This finding is consistent with another study that estimated the prevalence of obesity to be around 10%, with approximately 32% of obese individuals experiencing sleep disorders [27].

The bidirectional relationship between obesity and sleep disturbances has been extensively supported by numerous studies [10,27,28]. Obesity has been identified as a contributing factor to sleep disruptions through various mechanisms, while sleep disorders have been found to predispose individuals to weight gain and obesity. Recognizing and understanding these complex interrelationships is crucial when addressing both obesity and sleep health, as they have significant implications for the prevention and management of these conditions.

The accumulation of visceral adipose tissue in obese individuals may contribute to the secretion of inflammatory cytokines, which can disrupt the sleep-wake rhythm. Unhealthy dietary habits characterized by high consumption of fat and carbohydrates have also been linked to poorer sleep quality, while diets rich in fiber have been associated with more restorative and deeper sleep.

Experimental studies conducted in laboratory settings have demonstrated that reducing sleep duration or quality increases the risk of developing obesity, as it leads to physiological, hormonal, and behavioral changes that promote a positive energy balance [10].

While this particular study did not find significant differences in the prevalence of different sleep disorders among different BMI levels, a recent study conducted in China reported contrasting findings. The Chinese study revealed a significant association between insomnia and underweight individuals. Additionally, it reported increased odds of being underweight among insomnia patients with physiological hyperarousal [29]. These findings suggest that the relationship between sleep disorders and BMI may vary across different populations and should be further investigated.

The potential influence of medication use on sleep patterns is well-established [30]. However, in the current study, no significant association between sleep disorders and chronic medication use was found. This may be attributed to the demographic characteristics of the participants, who were predominantly younger individuals expected to be free from chronic conditions requiring medication.

The findings of this research have several implications for clinical practice and public health interventions. Healthcare providers should take into account the high prevalence of sleep disorders, particularly among younger age groups, unemployed/students, and obese individuals when evaluating patients. Screening for sleep disorders, early detection, and appropriate referral to sleep specialists can facilitate timely diagnosis and treatment. Public health campaigns and educational initiatives can play a crucial role in raising awareness about the importance of healthy sleep habits and the potential consequences of untreated sleep disorders.

The present study has several limitations that should be taken into consideration. Firstly, the validation of the Arabic version of the Sleep 50 questionnaire was conducted by the investigators themselves, which introduces the potential for bias. Additionally, the data on sleep quality relied solely on self-report measures, which may be subject to recall bias and inaccuracies. Furthermore, the study was conducted in a hospital-based setting, which may limit the generalizability of the findings to the broader population.

## Conclusions

In conclusion, the findings of this study indicate a high prevalence of sleep disorders among adults in Riyadh, Saudi Arabia. A significant proportion of individuals with sleep disorders were found to have multiple co-occurring sleep disorders, and there was a significant association with overweight and obesity. Early detection and appropriate interventions are crucial in order to improve the quality of life for these individuals and prevent further deterioration of their physical and psychological well-being.

## Figures and Tables

**Figure 4:**
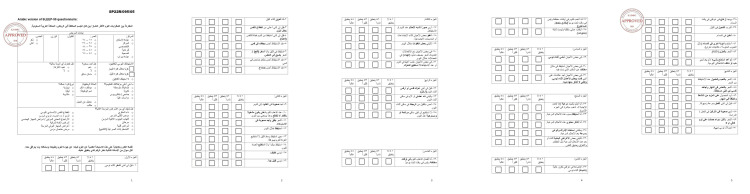
Arabic version of the questionarie

**Figure 5:**
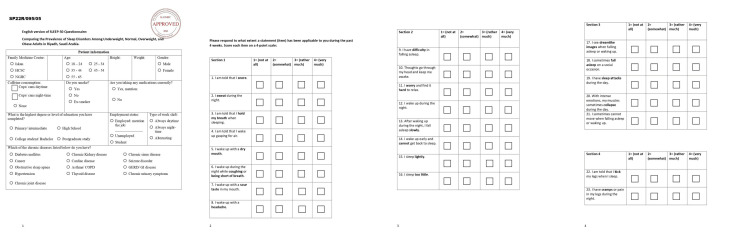
English version of questionnaire - part 1

**Figure 6:**
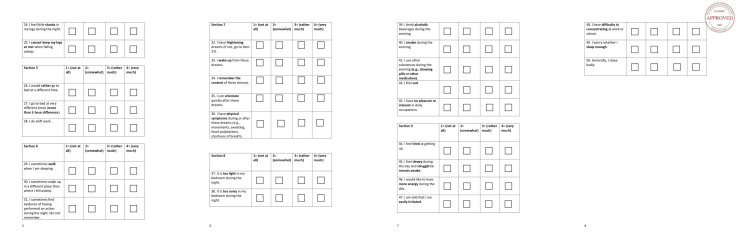
English version of questionnaire - part 2
